# Astrocytes: Role and Functions in Brain Pathologies

**DOI:** 10.3389/fphar.2019.01114

**Published:** 2019-09-27

**Authors:** Rosalba Siracusa, Roberta Fusco, Salvatore Cuzzocrea

**Affiliations:** ^1^Department of Chemical, Biological, Pharmaceutical and Environmental Science, University of Messina, Messina, Italy; ^2^Department of Pharmacological and Physiological Science, Saint Louis University School of Medicine, Saint Louis, MO, United States

**Keywords:** astrocytes, Alzheimer disease, Huntington disease, epilepsy, ischemic stroke, drug

## Abstract

Astrocytes are a population of cells with distinctive morphological and functional characteristics that differ within specific areas of the brain. Postnatally, astrocyte progenitors migrate to reach their brain area and related properties. They have a regulatory role of brain functions that are implicated in neurogenesis and synaptogenesis, controlling blood–brain barrier permeability and maintaining extracellular homeostasis. Mature astrocytes also express some genes enriched in cell progenitors, suggesting they can retain proliferative potential. Considering heterogeneity of cell population, it is not surprising that their disorders are related to a wide range of different neuro-pathologies. Brain diseases are characterized by the active inflammatory state of the astrocytes, which is usually described as up-regulation of glial fibrillary acidic protein (GFAP). In particular, the loss of astrocytes function as a result of cellular senescence could have implications for the neurodegenerative disorders, such as Alzheimer disease and Huntington disease, and for the aging brain. Astrocytes can also drive the induction and the progression of the inflammatory state due to their Ca^2+^ signals and that it is strongly related to the disease severity/state. Moreover, they contribute to the altered neuronal activity in several frontal cortex pathologies such as ischemic stroke and epilepsy. There, we describe the current knowledge pertaining to astrocytes’ role in brain pathologies and discuss the possibilities to target them as approach toward pharmacological therapies for neuro-pathologies.

## Introduction

During development, radial glial cells are the primary neural stem cells developing all neurons such as astrocytes, microglia cells, ependymal cells, and oligodendrocytes (Taverna et al., 2014). Mature astrocytes are categorized for functional and morphology proprieties. In the frontal cortex, these cells can be morphologically distinguished in four types: fibrous astroglia, protoplasmic, varicose, and interlaminar projections placed in the white matter and I, II, III, IV, V, and VI layers (Vasile et al., 2017). Other functional and morphological distinct astrocytes are unipolar Bergmann glia with radial ascending processes and elongated radial glia-like tanycytes. In the cerebellum, Bergmann glia control the synapsis of Purkinje cells (De Zeeuw and Hoogland, 2015), while in the hypothalamus, tanycytes are specialized in the modulation of neuroendocrine functions (Prevot et al., 2018). One of the most important astrocytes function is to deliver energy to neurons by the astrocyte-neuron lactate shuttle (Bass et al., 1971; Sherwood et al., 2006). Astrocytes modulate Ca^2+^ variations that influence neuronal activity releasing gliotransmitters (Peteri et al., 2019). The modulation of the neurotransmitter uptake involves the excitatory transporters 1 and 2 (EAAT1 and 2) (Roberts et al., 2014). In response to inflammation and injury, astrocytes become reactive. They can be divided in two main categories: scar-forming astrocytes and hypertrophic astrocytes (Khakh et al., 2017). Several studies underline that reactive astrocytes alter them homeostatic functions such as potassium ion uptake, ion buffering, Ca^2+^ signaling, and excitatory neurotransmitter uptake (Rossi and Volterra, 2009). Regulation of astrocytes functions affected several brain pathologies such as Alzheimer disease, Huntington disease, Ischemic stroke, and epilepsy.

## Alzheimer Disease

AD is a neurodegenerative disease with motor abnormalities, cognitive changes, and behavioral impairment. It is characterized by the aggregation of amyloid-β plaques in vessel walls and accumulation of the protein tau in neural cells. Astrocytes in this pathology contribute to the loss of neuroprotection and to the gaining of pathological characteristics. At the beginning, astrocytes have a protective role up-taking and degrading amyloid-β. The progression of disease leads to reduced astrocyte clearance of amyloid-β that contribute to gain of function (Garwood et al., 2017). Furthermore, amyloid-β accumulation stimulates astrocytes to produce pro-inflammatory mediators inducing a positive feedback of activation (González-Reyes et al., 2017).

It has been shown that amyloid-β co-operates with several receptors located on astrocytes such as scavenger receptors, TLRs, lipoprotein, glycoprotein and acetylcholine receptors, chemokine, and complement receptors (Farfara et al., 2008). Scavenger receptors are a group of evolutionally conserved membrane receptors expressed on the surface of microglia, macrophages, and dendritic cells (Wilkinson and El Khoury, 2012). To date, they have been classified into six classes (scavenger receptor A, B, C, D, E, and F) even if some members of this family remain unclassified (RAGE, CD163, and SR-PSOX). Of particular interest during AD are CD36, RAGE (receptor for advanced glycation end products), SCARA-1 (scavenger receptor A-1), and MARCO (macrophage scavenger receptor with collagenous structure). SCARA-1 is involved in clearance of Aβ, while MARCO forms a complex with formyl peptide–receptor-like 1 (FPR1) upon encountering Aβ. MARCO may decrease the inflammatory response in microglia through the FPR-1 *via* the ERK 1/2 intracellular signaling and the inhibition of cAMP (Brandenburg et al., 2010). CD36 and RAGE are implicated in activation of microglia by Aβ. CD36 cooperates with the other innate immune pattern recognition receptor like the TLRs to outline pathogen-specific responses. Once engaged by Aβ, CD36 forms a complex with TLR-6 and TLR-4 causing ROS production and inflammasome activation (Stewart et al., 2010). RAGE receptor is one of the most characterized unclassified scavenger receptor and has been reported to produce proinflammatory modifications in astrocytes when binds amyloid-β (González-Reyes et al., 2017). RAGE in turn activates the NF-κB (Yan et al., 1994) and its downstream pathway including p21, Cdc42-Rac, ras, MAPK (Taguchi et al., 2000), ERK (Wilkinson and El Khoury, 2012), and JNK (González-Reyes et al., 2017). RAGE is highly expressed vasculature and neurons in AD brains compared with the un-diseased (Arancio et al., 2004). RAGE located on endothelial cells in implicated in transporting Aβ into the brain (Mackic et al., 1998), and also increasing the diapedesis of monocytes across the blood–brain barrier (Giri et al., 2000). Once bound to soluble Aβ, RAGE induces microglial activation and chemotaxis following a concentration gradient, leading to a microglial accumulation around Aβ plaques (Wilkinson and El Khoury, 2012). RAGE mediates also the phagocytic profile of astrocytes and the interaction with other ligands, including S100β, involved in Alzheimer disease neuroinflammation (Cirillo et al., 2015). S100β produced by astrocytes is a common feature of Alzheimer disease (Bosch et al., 2015). It is associated with depressive behavior and cognitive flexibility and regulates neuronal oscillations (Stroth and Svenningsson, 2015; Brockett et al., 2018).

Moreover, morphological modifications of astrocytes in Alzheimer disease involve alterations in K^+^ neurovascular regulation, by downregulation of Kir4.1 and BK_Ca_, causing irregular cerebral blood flow (González-Reyes et al., 2017). Also, Ca^2+^ signaling is altered by amyloid-β accumulation (Haughey and Mattson, 2003). In astrocytes, this accumulation modifies the expression of the nicotinic acetylcholine receptors (nAchRs) and metabotropic glutamate receptor 5 (mGluR5), changing Ca^2+^ homeostasis (Xiu et al., 2005; Lim et al., 2013). Through this pathway, astrocytes increase glutamate signaling and led to the downregulation of its transporters (Masliah et al., 1996). Glutamate aberrant trafficking is linked to the modified cholesterol synthesis (Tian et al., 2010; Merlini et al., 2011; Talantova et al., 2013). A prodromal symptom to Alzheimer’s disease can be the glucose hypometabolism (Mosconi et al., 2006). Carriers of apolipoprotein Eε4 (APOEε4) allele display lower glucose metabolism in different brain area with an augmented risk for AD (Reiman et al., 2004). Astrocytes signaling is a useful target to prevent and control the development of the AD.

## Huntington Disease

Huntington disease is a genetic neurodegenerative disease with neuropsychiatric and motor dysfunctions. It is caused by a trinucleotide repeat (CAG) in the gene for Htt. This expansion caused a different form of Htt (mHtt) which aggregates (Bunner and Rebec, 2016). Astrocytes are more efficient than neurons in clearance of aggregates, so they are more resistant to mHtt accumulation (Zhao et al., 2016; Jansen et al., 2017; Zhao et al., 2017). However, when mHtt aggregates into astrocytes modifies glutamate signaling, causing neuronal excitotoxicity (Shin et al., 2005; Bradford et al., 2009). This condition is a typical feature of Huntington disease but has also been described several cases without alteration in glutamate release (Parsons et al., 2016). Astrocytes in Huntington disease are characterized by a decreased expression of Kir4.1 (Tong et al., 2014; Zhang et al., 2018). It influences GLT1-mediated homeostasis and Ca^2+^ signaling (Tong et al., 2014; Jiang et al., 2016). These dysfunctions head the reactive state of astrocytes bringing about the possibility neurotoxicity can induce inflammation as secondary effect of Huntington disease (Tong et al., 2014).

During the inflammatory state, microglia trigger the activation of astrocytes releasing factors such as TNF-α, C1q, and IL-1α (Khakh and Sofroniew, 2015; Liddelow et al., 2017). They decreased synaptic maintenance and phagocytic activity (Bradford et al., 2009) and increase degeneration neurons and oligodendrocytes (Liddelow et al., 2017).

mHtt accumulation modifies astrocytes exosome (Hong et al., 2017) and BDNF (Hong et al., 2016) release. Restoration of BDNF expression from astrocytes displays neuroprotective effects (Giralt et al., 2010; Hong et al., 2016; Reick et al., 2016). It has been displayed that astrocytes are intricated in a wide range of pathological features of Huntington disease, so they can be used as a novel therapeutic target.

## Epilepsy

Epilepsy is a group of brain disorders characterized by unpredictable and periodic occurrence of seizures. The cause of most cases of epilepsy is unknown. Some cases occur as the result of brain injury, stroke, brain tumors, infections of the brain, and birth defects through a process known as epileptogenesis (Goldberg and Coulter, 2013). Known genetic mutations are directly linked to a small proportion of cases (Pandolfo, 2011). Although the symptoms of a seizure may affect any part of the body, the electrical events that produce the symptoms occur in the brain. Epileptic seizures are the result of excessive and abnormal neuronal activity in the cortex of the brain (Fisher et al., 2005).

The most common of these pathologies is the hippocampal sclerosis or mesial temporal sclerosis. It is characterized by gliosis, neuronal cell loss in the hippocampal areas, synaptic reorganization, and microvascular proliferation. A study published in PloS Biology shows how the interaction between neurons and astrocytes is one of the mechanisms that contributes to the generation of epileptic discharges. Believed in the past to be simple “helpers” of neurons, astrocytes have revealed over time cells that play a much more active role in the brain (Gomez-Gonzalo et al., 2010). Astrocytes express ion channels, transmitter receptors, and transporters and, thus, are endowed with the machinery to sense and respond to neuronal activity. Glutamate transporters are located on several neuronal cell types, but astrocytes are mainly involved in the glutamate uptake (Steinhauser et al., 2016). GLT-1, the glutamate transporter located on astrocytes, is involved in the bulk of extracellular glutamate clearance and is responsible of the increased levels in epileptogenic foci. Moreover, glutamine synthetase is reduced in the hippocampus of temporal lobe epilepsy patients compared to the healthy one. This downregulation leads to a slow glutamate–glutamine cycling and an accumulation of the transmitter in the extracellular space and astrocytes, providing a metabolic mechanism for astrocyte-dependent hyperexcitability. A few studies have highlighted the contribution of ionotropic glutamate receptors in convulsion generation. AMPA receptors, in particular the subtype composed by subunits GluR1 to GluR4, are abundantly expressed on astrocytes. Epilepsy patients show an enhanced expression of GluR1 flip variants accounts for the prolonged receptor in hippocampal astrocytes. Prolonged receptor opening increases influx of Na^+^ and Ca^2+^ ions, blocking astroglial Kir channels which increase depolarization reducing the K^+^ buffering capacity of astrocytes (Steinhauser et al., 2012). All this process contributes to hyperexcitability. In this condition, extracellular [K^+^] could increase from ∼ 3 mM to 10–12 mM; and glial cells take the most K^+^ released by active neurons. As already mentioned, the primary mechanism for spatial K^+^ buffering and K^+^ reuptake is *via* glial inwardly rectifying K^+^ channels (Kir channels). Kir channel subtypes (Kir1–Kir7) differ in functional properties and tissue distribution; Kir4.1 is the most abundantly in brain astrocytes. Astrocytes are also joined by gap junctions, which allow these cells to redistribute through the glial network the K^+^ ions excessively accumulated at sites of intense neuronal activity. Accordingly, increasing evidence indicates that dysfunctional astrocytes are crucially involved in processes leading to epilepsy (Steinhauser and Seifert, 2012).

## Ischemic Stroke

Ischemic stroke is a brain damage which can lead to death or disabilities. It results from a vasculature dysfunction with occlusion of blood vessels by embolus or thrombus. The reduced or blocked blood flow causes loss of oxygen and glucose and in turn synthesis of ATP *via* glycolysis and oxidative phosphorylation. These conditions produce excitotoxicity and malfunction of astrocytes glutamate transporters, fundamental in the synaptic cleft in clearing glutamate release (Yi and Hazell, 2006; Zou et al., 2010). Increased glutamate release in the extracellular area induces the overexpression of rNMDARs and caused overloading of intracellular Ca^2+^ (Tanaka et al., 1997; Medvedeva et al., 2009). This energy depletion influences membrane potential depolarization and ionic gradients in neurons and astrocytes. In particular, astrocytes, comparing neurons, are less susceptible to glutamate cytotoxicity induced by brain stroke, but they display proliferation and up-regulation of GFAP levels producing reactive astrogliosis (Sofroniew, 2000). Reactive astrocytes are usually found in the focal lesions with tissues reorganization and formation of glial scars (Sofroniew, 2000). White matter astrocytes are especially sensible to ischemic stroke (Chen et al., 2016). The ischemic core shows a predominant presence of hypertrophic astrocytes with a larger Ca^2+^ signal compared to the penumbra region, the area surrounding the ischemic locus (Ding et al., 2009). Transcriptome analysis of activated astrocytes from inflamed brain after middle cerebral artery occlusion shows expression of genes encoding neuroprotective mediators and included cytokines (IL-6, IL-1, IL-1β, IL-10), transforming growth factor-β (TGFβ), interferon-γ (IFN-γ), thrombospondins, and neurotrophic factors (Zamanian et al., 2012). High levels of cytokines induce increasing levels of nitric oxide (NO) (Stoll et al., 1998) and apoptosis of neuronal cells (Clark and Lutsep, 2001) and inhibit neurogenesis (Monje et al., 2003). Reactive astrocytes also release chemokines after ischemia (Kim, 1996). In vascular endothelial cells, chemokines increased adhesion molecules levels, attracting immune cells (Sofroniew, 2000). Astrocytes are the first cells of the nervous system where the class II major histocompatibility complex (MHC) (Dong and Benveniste, 2001) was shown. MHC II presents antigens to CD41 T-helper cells and is expressed on antigen presenting cells (APCs). Moreover, astrocytes express pattern recognition receptors (PRRs) as scavenger receptor, TLRs, and complement proteins playing a role in immune response regulation (Bsibsi et al., 2006).

These features let us to consider astrocyte a possible regulator of the ischemic context, considering that chronic of inflammation is influenced by the degree of tissue injury and exacerbation of the damage.

## Discussion

To date, only five drugs are accepted by the Food and Drug Administration (FDA) for the cure of AD: donepezil, galantamine and rivastigmine, memantine, and a drug composed of donezil and memantine (**Table 1**). Unfortunately, the use of these drugs is aimed at improving the excellence of life of patients, and they are not capable to stop the progression of the disorder (Caselli et al., 2017). So, it is important to find innovative treatments that improve therapeutic results. Aβ plaques increase the proinflammatory cytokines (Patel et al., 2005; Colombo and Farina, 2016) and the production of free radicals (Carson et al., 2006; Wyss-Coray and Rogers, 2012) with consequent activation of the astrocytes. In a late study conducted on APP/PS1 transgenic mice and on mixed neuronal/glial cultures, it was shown that curcumin improves spatial memory, stimulates cholinergic neuronal function, and, through PPAR-γ, reduces the activation of the inflammatory process in microglia and astrocytes (González-Reyes et al., 2017). Additional natural phytochemicals have demonstrated an anti-inflammatory and immunosuppressive capacities in AD models (**Table 1**), e.g., the triptolide extract inhibits astrocyte activation in the APP/PS1 transgenic mouse model of AD (Li et al., 2016). Punicalagin, a pomegranate derivative, reduces neuroinflammation (lowering TNF-α and IL-β) and also prevents oxidative stress by reducing iNOS, COX-2, and ROS production (Kim et al., 2017). Other mixtures that may have a probable role against dementia (Libro et al., 2016) are cannabinoid agonists such as WIN, 2-AG, and methanandamide (**Table 1**) that have shown anti-inflammatory activities in primary astrocytes grown later exposure to Aβ_1–42_ or Aβ_25–35_ (Aguirre-Rueda et al., 2015; Gajardo-Gomez et al., 2017). Other approaches to diminish oxidative stress in AD models involve stimulants of endogenous antioxidant factors (**Table 1**) such as pelargonidine (Sohanaki et al., 2016), Bambusae concretio Silicea (Jeong et al., 2005), and the new compound Monascin (Shi et al., 2016). In *in vivo* and in *in vitro* analyses, it has been shown that exogenous antioxidant compounds (**Table 1**) also have beneficial effects. Among these, we have resveratrol (Wang et al., 2016), tocotrienol (vitamin E) (Ibrahim et al., 2017), anthocyanins (Rehman et al., 2017), epicatechin (Cuevas et al., 2009), and 3H-1,2-dithiole-3-thione (a powerful free radical scavenger) (Wang et al., 2017). Aβ accumulation from astrocytes can also be decreased using IL-1β or TNF-α/TNF-α, PPAR-γ receptor agonists, minocycline or nicergoline, and tyrosine kinase inhibitors (Von Bernhardi et al., 2010; Kitazawa et al., 2011; Mandrekar-Colucci et al., 2012; Tweedie et al., 2012). NSAIDs are drugs that bind to and activate the PPAR-γ receptor (Jaradat et al., 2001; Wick et al., 2002) leading to reduced activation of glial cells (Combs et al., 2000; Bernardo and Minghetti, 2006) and cytokine-mediated inflammation (Sastre and Gentleman, 2010; De Nuccio et al., 2015).

**Table 1:** Neurologically active drugs.

Disease	Drug category	References
AD	FDA accepted	Donepezil, galantamine, rivastigmine, memantine, and donezil + memantine (Caselli et al., 2017)
	Natural phytochemicals	Triptolide extract (Li et al., 2016) and punicalagin (Kim et al., 2017)
	Cannabinoid agonists	WIN, 2-AG, and methanandamide (Aguirre-Rueda et al., 2015; Gajardo-Gomez et al., 2017)
	Endogenous antioxidant factors	Pelargonidine (Sohanaki et al., 2016), Bambusae concretio Salicea (Jeong et al., 2005), monascin (Shi et al., 2016)
	Exogenous antioxidant compounds	Resveratrol (Wang et al., 2016), tocotrienol (Ibrahim et al., 2017), anthocyanins (Rehman et al., 2017), epicatechin (Cuevas et al., 2009), and 3H-1,2-dithiole-3-thione (Wang et al., 2017)
	Stimulators of the GLT1 expression	Penicillin, cephalosporin, ampicillin, estrogen, riluzole, and insulin (Frizzo et al., 2004; Brann et al., 2007; Ji et al., 2011)
	Activators of the GLT1 translation	Pyridazine and LDN/OSU-0212320 (Colton et al., 2010; Xing et al., 2011)
	GABA receptor antagonists	(Yuan and Shan, 2014)
Epilepsy	AED	Valproic acid, lamotrigine, phenobarbital, gabapentin, felbamate, and topiramate (French and Gazzola, 2011)
	Anticancer drug	Rapamycin (Huang et al., 2010; Kim and Lee, 2019)
	Allosteric potentiators of glutamine synthetase, regulators of AQP4 trafficking, interleukin 1 antagonists, and agonists or allosteric potentiators of TNFR2	(Wetherington et al., 2008) (Crunelli et al., 2015)
Ischemic stroke	Stimulators of the GLT1 expression	Ceftriaxone (Ouyang et al., 2007; Verma et al., 2010), carnosine (Shen et al., 2010), and tamoxifen (Lee et al., 2009)
	Inhibitors of p53 activity	MicroRNA-29a (Ouyang et al., 2013; Ouyang et al., 2014)
	Stimulators of angiogenesis	Ecdysterone (Luo et al., 2011) and omega-3 polyunsaturated fatty acids (Wang et al., 2014)

The astrocyte carries most of the extracellular glutamate. Therefore, damage to astrocytes affects their capability to perceive or respond to an increase in glutamate levels which leads to the destruction of the microenvironment near neurons causing an over-stimulation of NMDA receptors, responsible for changes in cognitive functions in the frontal cortex (Finsterwald et al., 2015). Current studies have shown that the damage to astrocytes induced by Aβ is responsible for the reduced expression of GLT1 in AD. Therefore, drugs that target astrocytic glutamate transporters to ameliorate their expression and role represent a possible target for neurodegenerative syndromes. In this regard, there are two pharmacological approaches to increase GLT expression: either by increasing GLT1 promoter activation or by activating GLT1 translation (Rothstein et al., 2005; Kong et al., 2014). Among the compounds able to stimulate the expression of GLT1 already 48h after drug treatment, there are β-lactam antibiotics comprising penicillin and its derivatives, as well as cephalosporin antibiotics. Other mixtures such as ampicillin, estrogen, riluzole, and insulin have also been found to increase GLT1 expression (Frizzo et al., 2004; Brann et al., 2007; Ji et al., 2011) (**Table 1**). Instead, among compounds that have been found to activate the GLT1 translation (**Table 1**), we have a series of compounds based on pyridazine and LDN/OSU-0212320 (Colton et al., 2010; Xing et al., 2011). Finally, recent studies have correlated GABAergic neurotransmission with the pathological changes of AD (Li et al., 2011). Damaged astrocytes produce a copious amount of GABA that is released to inhibit excitatory neurotransmission in the dentate gyrus. In addition to GABA, monoamine oxidase-B (MAO-B) has been reported to be altered on reactive astrocytes (Jo et al., 2014), and the enzyme is upregulated in the *post mortem* brain of individuals with AD (Saura et al., 1994). In an animal model of Alzheimer, it has been shown that the administration of GABA receptor antagonists (**Table 1**) improve long-term memory in the hippocampus (Yuan and Shan, 2014).

HD is a disease that progressively destroys neurons in the brain and leads to severe motor and cognitive deficits. To date, no cure is available, but researchers have made progress that can lead to effective therapies. Numerous studies suggest that astrocytes may be intricated in HD. In particular, it has been observed that mHTT accumulations in striatal astrocytes are present in the brains of HD patients and in HD mouse models (Bradford et al., 2009). Several HD mouse models have been used to evaluate the contribution of astrocytes to HD pathophysiology. In one of these studies, astrogliosis was evaluated as it frequently accompanies brain disorders. In conjunction with the start of symptoms, a high number of astrocytes showed mHTT inclusions and an important reduction in fundamental functional proteins. One of these proteins was Kir4.1 (Tong et al., 2014). These results propose that mHTT is correlated with early termination of the expression of essential functional astrocyte proteins (e.g., Kir4.1), which modifies the function of astrocytes without triggering astrogliosis. Furthermore, striatal astrocytes of HD mice show depolarized membrane potentials and lower membrane conductances when mice are symptomatic. This is owing to the function and lower expression of the Kir4.1 channels. Deficiencies in latent membrane potential were recovered by selective release of Kir4.1 from adeno-associated viruses (AAV) and a specific astrocyte promoter. Furthermore, it has been observed that the loss of Kir4.1 currents in striatal astrocytes leads to reduced K^+^ spatial buffering, which leads to higher environmental K^+^ levels in HD mouse models. Therefore, the astrocytic channels Kir.4.1, and other astrocytic molecular mechanisms can represent appreciated targets for therapeutic development (Khakh and Sofroniew, 2014).

Other approaches currently being studied for HD therapy point to both to obtain information on the mechanisms of disease progression and to silence the expression of mHTT using antisense oligonucleotides. A new approach is to detect novel factors that increase neurogenesis and/or stimulate the reprogramming of endogenous neuroblasts and parenchymal astrocytes to produce new healthy neurons to substitute the lost ones and/or strengthen the neuroprotection of preexisting striatal and cortical neurons (Sassone et al., 2018).

Regarding epilepsy, to date, more than 20 antiepileptic drugs (AEDs) (**Table1**) have been developed, including valproic acid, lamotrigine, phenobarbital, gabapentin, felbamate, and topiramate (French and Gazzola, 2011). Despite this, ∼30% of patients respond poorly to treatment (Kwan and Brodie, 2000). In contrast, 70% of patients can attain long-term remission under AED treatment. However, many AEDs are associated with adverse side effects that are experienced by a substantial number of patients. Thus, significant unmet medical needs still must be overcome for the real and safe treatment of epilepsy. Many studies have suggested that inequities between excitatory and inhibitory signals may cause epilepsy (White et al., 2007; Bialer and White, 2010). AEDs currently used to stop epileptic seizures act mostly by blocking ion channels and inhibiting neuronal excitability. Rapamycin, which was approved by the FDA as an anticancer drug (**Table 1**), has been demonstrated as another potential antiepileptic agent with broader clinical relevance (Huang et al., 2010; Kim and Lee, 2019). Unfortunately, rapamycin can inhibit cell proliferation and motility; thus, the safety of long-term rapamycin treatments must be assessed in advance. However, the role of the mTOR inhibition strategy for the treatment of epilepsy remains viable (Russo et al., 2014). Today, it is clear that astrocytes play prominent roles in information processing in the epileptic brain. Insights gleaned from careful studies of the properties of reactive astrocytes suggest several novel targets for drug development (**Table 1**), including allosteric potentiators of glutamine synthetase, regulators of AQP4 trafficking, interleukin 1 antagonists, and agonists or allosteric potentiators of TNFR2 (Wetherington et al., 2008) (Crunelli et al., 2015).

To date, pharmacological treatments for ischemia/reperfusion have palliative effects and require almost immediate administration after damage (Van Der Worp and Van Gijn, 2007). To overcome this problem, it is indispensable to find new treatments focused mainly on long-term neuroprotection. Strategies targeting astrocytes may be an option as the increase in astrocyte survival during ischemic stress is connected with increased neuronal survival. It has been observed that induction of glial-specific purinergic receptor activation (P2Y1R) leads to greater consumption of mitochondrial O_2_ and stimulation of ATP production by astrocytes thus reducing neuronal damage to astrocytes and cell death and therefore brain damage (Zheng et al., 2013; Liu and Chopp, 2016). Furthermore, infarct area improved even after administration of TGF-α (Sharif et al., 2007). This treatment also led to a significant functional recovery in rats after MCAO (Justicia et al., 2001). Other experiments indicate that another therapeutic potential involves the increase in astrocytic glutamate transport after stroke. Thus, the increased expression of the glutamate transporter GLT-1 in astrocytes with ceftriaxone (**Table 1**) (Ouyang et al., 2007; Verma et al., 2010) protects neurons from ischemia (Chu et al., 2007). Other compounds that improve neurological function and reduce the infarct area are carnosine (Shen et al., 2010) and tamoxifen (Lee et al., 2009) (**Table 1**). Both substances preserve the expression of GLT-1 on astrocytes by reducing glutamate levels and attenuating the consequent excitotoxicity. Another target for stroke therapy is p53 (**Table 1**) since inhibition of p53 activity has been shown to hinder astrocyte activation and glutamate intake (Ahn et al., 2015). Even microRNAs, approximately of which are expressed in astrocytes as microRNA-29a, appear to be intricate in the control of cerebral ischemia and may represent targets for improving stroke outcome (Ouyang et al., 2013; Ouyang et al., 2014). More recently, reference is made to cell therapy which aims at finding cells that can induce regeneration. Astrocyte transplantation conducts to recovery of axonal myelination, variation of the immune response, and issue of neurotrophic factors that prevent oxidative stress and excitotoxic injury (Choudhury and Ding, 2016). Other studies have suggested to astrocytes a therapeutic target based on their control by genetic change of proteins associated to the immune response and exacerbation of reactivity and cytotoxicity (Merienne et al., 2015). Finally, it was observed that post-stroke angiogenesis not only ameliorate blood perfusion in the ischemic area but also supports cerebral parenchymal cells, comprising astrocytes, the issue of neurotrophic factors, to stimulate neurogenesis, which therefore improves remodeling cerebral and long-term neurological function after stroke (Zhang and Chopp, 2009). Consequently, angiogenesis represents a valid reparative machinery that has been verified in numerous studies (**Table 1**). For example, treatment with ecdysterone ameliorates neurological function by improving astrocyte stimulation and angiogenesis after focal cerebral ischemia in rats (Luo et al., 2011). Transgenic overproduction of omega-3 polyunsaturated fatty acids in mice recovers post-stroke revascularization and increases endogenous angiogenesis by inducing angiopoietin 2 production in astrocytes, which consequently stimulated endothelial cell proliferation and BBB formation, proposing that the integration of omega-3 polyunsaturated fatty acids is a possible angiogenic treatment able to increase brain repair and improve long-term functional recovery after ischemic stroke (Wang et al., 2014).

## Author Contributions

RS and RF made literature search and wrote the first draft of the manuscript. SC, RS and RF designed the aim of the review. All authors contributed to reading and approving the final version of the manuscript.

## Conflict of Interest

The authors declare that the research was conducted in the absence of any commercial or financial relationships that could be construed as a potential conflict of interest.
